# Hydraulic Fracturing Design Considerations and Optimal Usage of Water Resources for Middle Eastern Tight Gas Reservoirs

**DOI:** 10.1021/acsomega.1c01602

**Published:** 2021-05-14

**Authors:** Abhijith Suboyin, Md Motiur Rahman, Mohammed Haroun

**Affiliations:** Department of Petroleum Engineering, Khalifa University of Science and Technology, Abu Dhabi 2533, United Arab Emirates

## Abstract

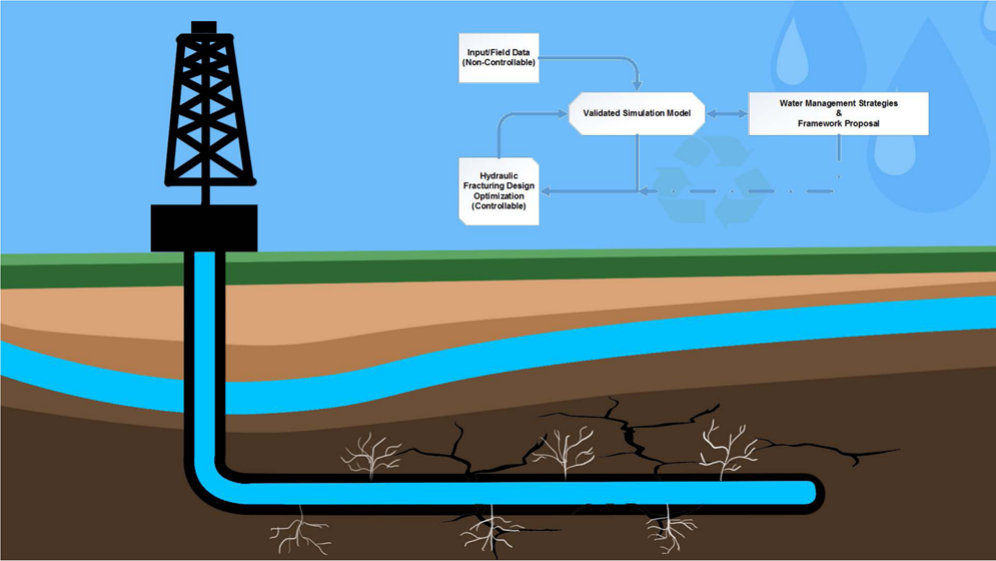

Over the past few decades, hydraulic fracturing, a well-stimulation technique commonly used for extracting hydrocarbons within unconventional reservoirs, has played a significant role in transforming the energy industry. Multiple studies and field trials have proven that an effective, efficient, and economical approach is critical for such operations. However, even after numerous fracturing jobs conducted across the globe, they are still related with high risk. Moreover, the exploitation of such reservoirs is water- and resource-intensive as compared to conventional reservoirs. This is crucial, especially in offshore operations and arid regions. A comprehensive investigation through a traditional fracture design process was conducted for a candidate Middle Eastern reservoir. Through the construction of strategically constrained cases in the presence of complex natural fracture sets, this novel investigation allowed the model to successfully isolate and characterize the key fracture design parameters that influenced fracture geometry for the candidate field and in turn the requirements with respect to water usage and resource consumption. The results indicate that for the given field conditions, fluid and proppant optimization is critical to achieving maximum recovery. The influence of natural fracture is highly critical and greatly influences the overall productivity. Simulations further indicate water requirements for the candidate field ranging from 3.5 to 5.8 million gallons of water per operation, which is significant in water-scarce regions. The findings of this study and the proposed workflow can assist to better understand the distinct contributions of key fracture design and operational parameters that are critical under the current volatile market conditions.

## 1 Introduction

Water is often regarded as a prime commodity essential for livelihood and vital for the survival and development of all natural life. Even so, it is reported that a considerable number of the human population currently lives in water-scarce areas. The United Nations relates water scarcity as scarcity in availability due to physical shortage or scarcity in access due to the failure of institutions to ensure a regular supply or due to a lack of adequate infrastructure. It further reports that over 2 billion people live in countries experiencing high water stress and estimates that, by 2040, one in four of the world’s children (under the age of 18) will be living in areas of extremely high water stress.^1^

Since the 19th century, oil and gas has also become an essential commodity, which has been contributing significantly to the development of the world economy. However, the petroleum industry is heavily dependent on water resources, and water management is at the core of sustainable development for the industry. Along with population growth coupled with rapid urbanization, it is reported that energy requirements are predicted to grow up to 55% by 2030. This places additional stress on water resources and additionally increases water demand.^2^

With the hydrocarbon industry, extraction of water resources is extremely limited, sensitive, and governed by multiple regulations, which are regional. Hence, there has been an increased interest for efficient, economic, and environment-friendly operations. This plays a significant role in water-scarce and arid regions such as the Middle East.

Under traditional conventional resources, the values for porosity and permeability are high enough for the formation to produce naturally, especially without any external well stimulation induced. Conversely, in the case of unconventional reservoirs, the fluids are more constrained as they are trapped inside tiny pore spaces due to the extremely low permeable nature of the formation. Among these resources, it is reported that shale and tight (low permeable) reservoirs may particularly play a central role and contribute considerably to the world’s cumulative energy production.^3,4^

Hydraulic fracturing has been proven as one of the most practicable solutions to tap into such resources. Often termed as “fracking,” this well-stimulation technique involves injecting a combination of liquid, sand, polymer, and/or chemicals under extraordinary pressure into bedrocks. As a result, this process allows enhanced hydrocarbon flow from low-permeability rocks such as shales, tight sandstone, or coal beds. Multiple studies have tried to accurately capture fluids within fracture under a tight porous media, and there have been significant advancements regarding the same.^5^ In the current volatile market condition, ascertaining uncertainties and advancing current practices within the petroleum industry can provide significant gains for an operator.

**Figure 1:**
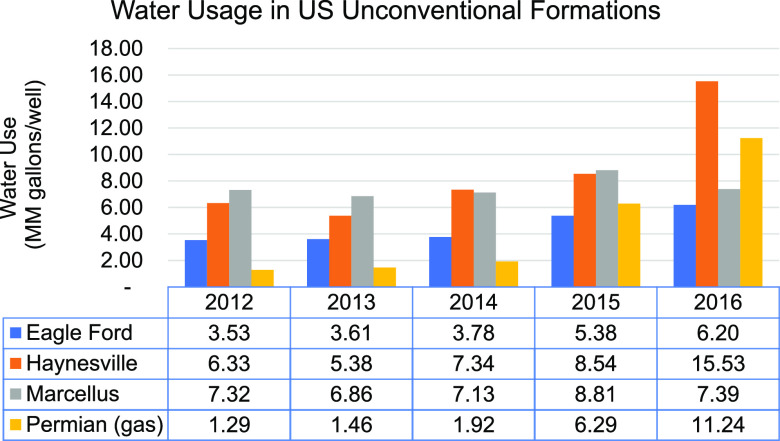
Water consumption within major shale gas plays in the United States.

Figure 1 illustrates water consumption within major shale gas plays in the United States.^6^ Multiple studies have already investigated the intensification of water usage in such plays, and the literature has also reported that a representative production well within the United States, over its life cycle, may consume as much as 84 MM gallons of water.^6,7^ However, these estimated figures are also dependent on multiple variables such as the targeted number of fractures for a given well, reservoir characterization, refracturing operations, etc. Studies have also shown how a significant volume of the injected water may be left behind, subsequently affecting the formation along with potential reservoir damage.^7^ Furthermore, even after effective stimulation of such formations, it is often reported that the targeted geometry, permeability, and efficiency are often not achieved. In addition, there are complications in modeling the accurate flow behavior of hydrocarbons and other fluids in reservoirs where fractures exist. The presence of such fractures often leads to complications such as premature water breakthroughs, reduced recovery rates, channeling of injected fluids, and fracture collapse due to changes in reservoir pressure.^8^ As a result, these lead to higher costs or low ultimate recoveries, which are critical parameters to be considered in the current market conditions.

Even with multiple successful field cases in unconventional formations across the globe, development of such reservoirs is still related to a high degree of uncertainty.^4,8,9^ With the ever-increasing energy demand and advancements in governing policies with respect to environmental resources, it is crucial that we enhance the current methodologies in place while targeting our deliverables. As a significant amount of resources (time, cost, and personnel) is involved, it is critical to identify potential strategic drivers along with potential concerns and challenges within the transformational regional market such as the Middle East.

This comprehensive investigation is an extension of the study conducted by Suboyin et al.^9^ The preliminary model and previous investigations provided an insight into hydraulic fracturing design consideration in highly naturally fractured reservoirs within the Middle East. This extended investigation presents quantitative characterization of hydraulic fracturing treatment design parameters along with potential design considerations, particularly for sustainable resource management for Middle Eastern tight gas reservoirs. Key advantages for such an investigation include identifying approaches for efficient management of resources in arid regions while evaluating existing water-management strategies, global applications along with their justification, and potential application in arid/water-scarce regions such as the Middle East.

The model used within this investigation is based on a candidate Middle Eastern tight gas reservoir, and the influences of key parameters were analyzed through the construction of simplistic constrained cases. This included categorizing the parameters as controllable and noncontrollable parameters, further elaborated in a later section of this study. These constrained cases allowed one to successfully analyze the distinct contribution of each parameter to the overall productivity and the success of the hydraulic fracturing operation. For this investigation, the water requirement for typical fracturing operations was analyzed in detail by varying a singular parameter while keeping other design parameters constant. This allowed the model to successfully isolate and characterize the key fracture design parameters that influenced fracture geometry for the candidate field and in turn the requirements with respect to water usage. This resulted in water requirements for operations for the candidate field ranging from 3.5 to 5.8 million gallons of water per operation depending on the fracturing design, which is significant in water-scarce regions.

Within this investigation, the research objectives and motivation are presented first. This is followed by the methodology, where the current techniques are discussed in brief prior to the introduction of the constructed model, the fundamental underlying equations, and the input data for the candidate field case study. Furthermore, the results are analyzed and further discussed in depth along with presenting a parametric sensitivity analysis. This leads to the proposed workflow based on key findings and outcomes from this investigation along with regional data. The conclusions are summarized toward the end, and an Appendix 1 is provided to further understand the underlying equations of the model.

**Figure 2:**
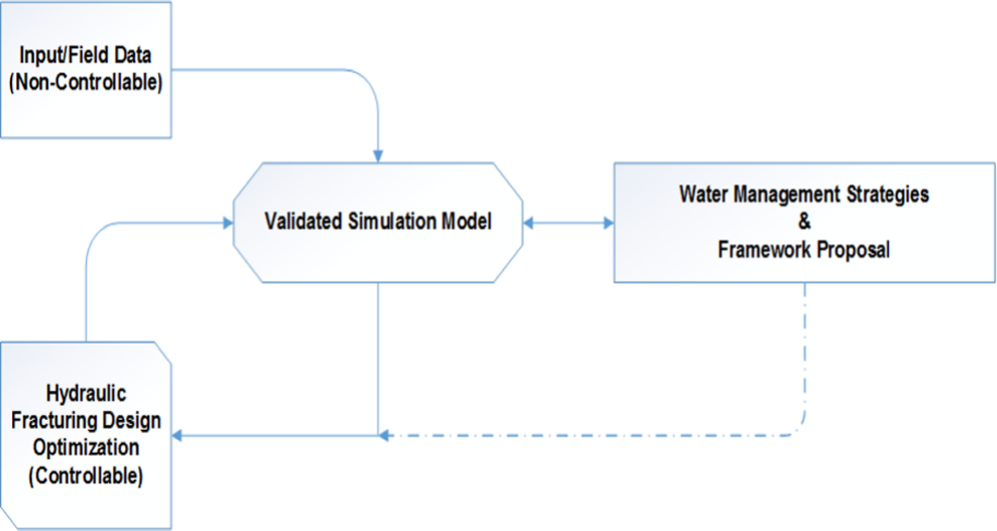
Investigation workflow: overview.

A brief workflow of the overall process is given in Figure 2. Based on an iterative process, the validated simulated model is based on controllable and noncontrollable input data. Once analyzed, the controllable parameters are further examined and optimized based on the target objectives. Upon successful accomplishment of the target objectives, the key parameters are investigated to identify the optimal strategies for the region along with a proposal of the framework. This can be further fed back into the model to further optimize the model and the controllable input parameters for fit-for-purpose objectives and tailored strategies.

Hence, the key objectives for this research are as follows:

(1) Examine and analyze hydraulic fracture propagation for a candidate Middle Eastern tight gas reservoir in the presence of complex natural fractures.
(2) Advance an adaptable simulator model to examine, identify, and quantitatively characterize the dominant fracture design parameters for the given reservoir conditions.
(3) Conduct a sensitivity analysis to identify key parameters influencing fracture geometry along with identifying potential design considerations and improve efficiency with respect to resource management.
(4) Propose a unique operational and sustainable workflow to highlight the governing parameters for efficient water-management strategies for arid regions such as the Middle East.

## 2 Methodology

There have been considerable advancements in completion methods with respect to fracturing domain over the past few decades. Frac and pack, hydra-jet perforation, zipper fracking, proppant selection, fracturing fluid optimization, and fracture mapping coupled with microseismicity are some technologies that aided in the economical and efficient recovery from tight reservoirs.^10−15^

Studies mainly relate the fundamentals of fracture instigation, propagation, and analysis to in situ stresses mainly regarded as three components, namely, compressive, isotropic, and nonhomogenous stresses. Studies have shown how factors such as overburden, pore pressure, formation properties, temperature, diagenesis, tectonics, etc. greatly influence these stresses, and a fracture is created in a direction perpendicular to the minimum stress.^16^

**Figure 3:**
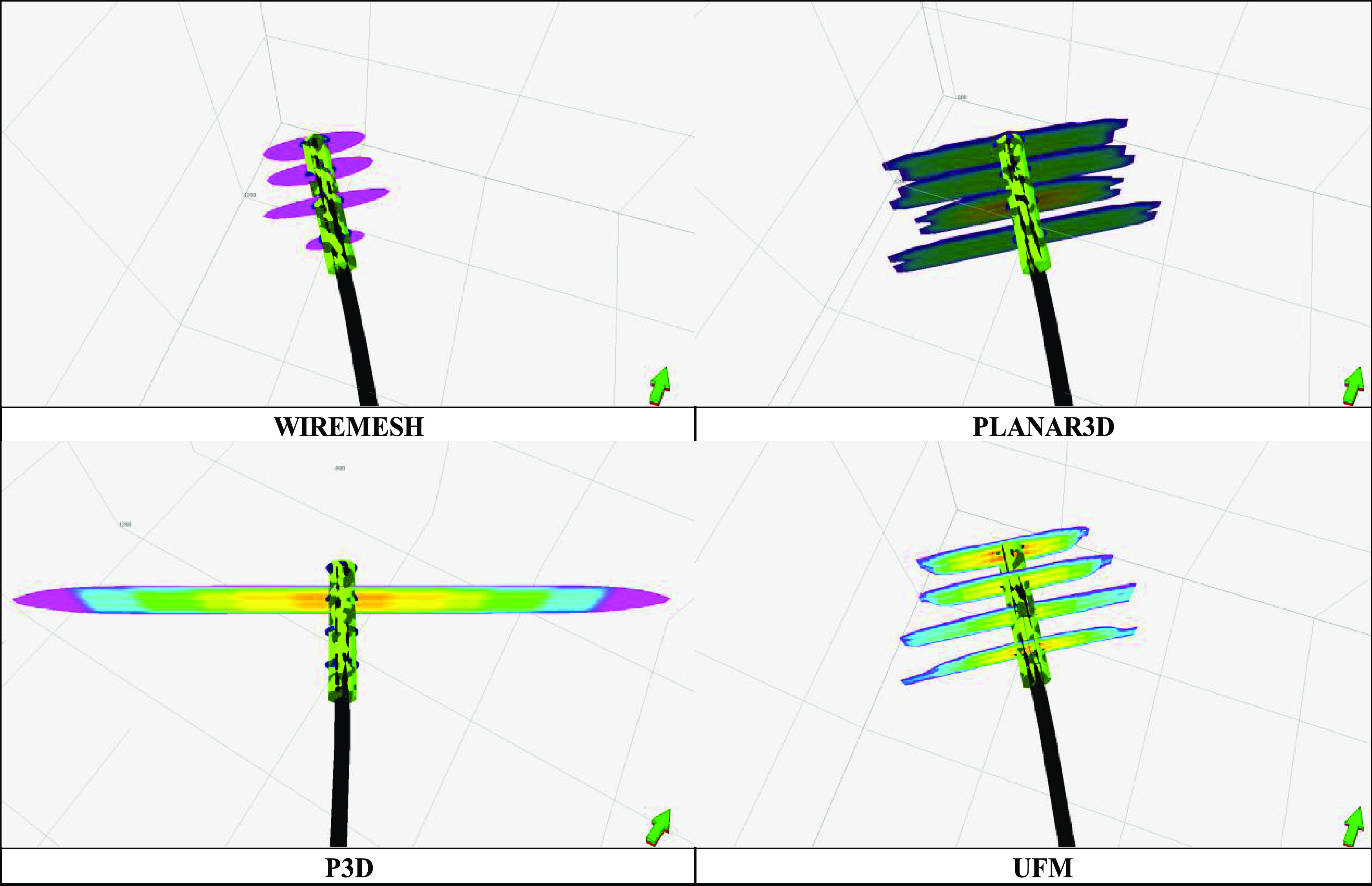
Fracture models compared within the simulator. Reprinted with permission from ref (9). Copyright 2020 Elsevier.

Numerous modeling techniques were proposed for prediction of fracture geometry and productivity. As shown in Figure 3, Wiremesh, Planar3D, Pseudo 3D, and the unconventional fracture model (UFM) are a few of the extensively implemented approaches within the industry, with each having its own advantages and constraints. It is evident that interaction of natural fractures with hydraulic fractures can lead to fracture growth and propagation.^17,18^ Parameters such as the stress distribution, reservoir heterogeneity, and natural fracture distribution/orientation are reported to play a significant role in the same. UFM, a model recently developed, incorporates the stress fields, natural fracture orientation, and rock deformation that are critical to analyzing the hydraulic fracture propagation behavior in an unstructured grid.^19^ Hence, for this investigation, UFM was selected.

Multiple solutions exist in the industry for modeling the flow behavior and mass transport within porous media. The most notable among them are the dual-continuum method (DCM) and discrete fracture networks (DFNs). Under the DCM, the matrix and fracture are modeled as two separate continua possessing the same control element or volume with respect to space.^20^ However, since the geometry of the discrete fractures is not explicitly modeled along with the solution or flow pathway, they result in erroneous flow calculations in reservoir portions where well control is restricted.^21^ In contrast, the DFN model solves some of these shortcomings as it involves analysis and modeling, which explicitly incorporates the geometry and properties of discrete features as a central component controlling flow and transport.^18^ DFNs can lead to a more realistic description of the network, as they are stochastic models that incorporate statistical scaling rules derived from the analysis of fracture length, height, spacing, orientation, and aperture.^22^

This investigation began with the creation of a rudimentary simulation case to analyze the fundamentals of hydraulic fracture propagation. A complex natural fracture set was introduced to the system, and the system behavior was further evaluated. An in-depth literature review was conducted to understand the applications of industrial simulators in tight gas reservoirs along with data acquisition for the preliminary models. This was followed by creating comprehensive models using field data and validation. The interaction, the fracture propagation behavior, and the production pertaining to variations in fracture design parameters along with interaction with natural fractures were also studied. This was extended by building a realistic model based on field data along with history matching.

One of the commercial simulators used for this investigation has the capability to model three-dimensional (3D) hydraulically induced fracture propagation in unconventional reservoirs with ultralow permeability along with discrete fracture networks. As per the literature,^23−25^ the equation for mass conservation for an incompressible slurry that is to be pumped into the fracture can be represented as eq 1.
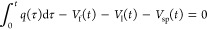
1wherein *q* denotes the general injection flow rate, *t* is the time required for fracture leak-off area creation, *V*_f_ is the volume of fracture, *V*_l_ is the fluid loss, and *V*_sp_ is the spurt loss. For any hydraulic fracturing treatment, it is critical to consider leak-off fluid loss, pre- and postpumping. This is expressed as eqs 2 and 3(25)
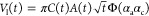
2
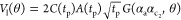
3wherein α_a_ denotes the leak-off parameter, α_ζ_ is the leak-off parameter (fracture), α_c_ is the leak-off parameter (pumping), α_c_2__ is the reservoir compressibility and viscosity coefficient, Φ is porosity, and θ is the dimensionless time. Studies further report that the relationship between fracture opening and pressure can be written as eq 4(26)
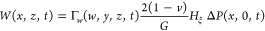
4where Γ denotes the generalized function related to influence and *G* is the generalized function related to fluid loss. These are the fundamental equations that need to be considered, and further underlying equations are provided in the Appendix 1.

**Figure 4:**
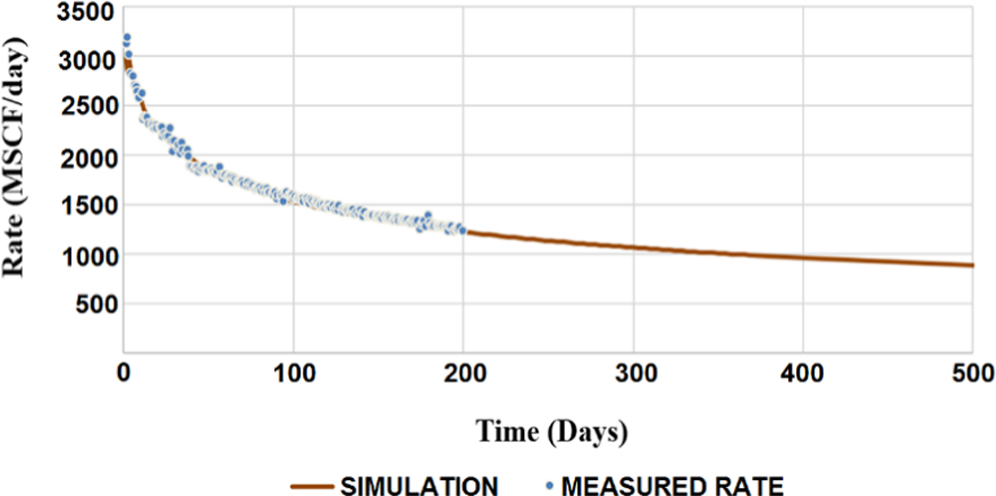
History matching of the constructed model. Reprinted with permission from ref (9). Copyright 2020 Elsevier.

Based on an iterative process and these central equations discussed, the results of the constructed simulation models are analyzed in depth with respect to the input data. The constructed model consisted of a reservoir model and a fracture model to effectively identify the contribution of each parameter, as they are extremely codependent. The validation of the constructed model was also accomplished by comparing the simulation results with data from candidate field and literature. For instance, the history match of the production data, i.e., the production rate (measured) vs the rate of fracture flow (simulated), is shown in Figure 4.^9^ The history match of the production data, i.e., the production rate (measured) vs the rate of fracture flow (simulated), helps us further verify the simulation results.

**Figure 5:**
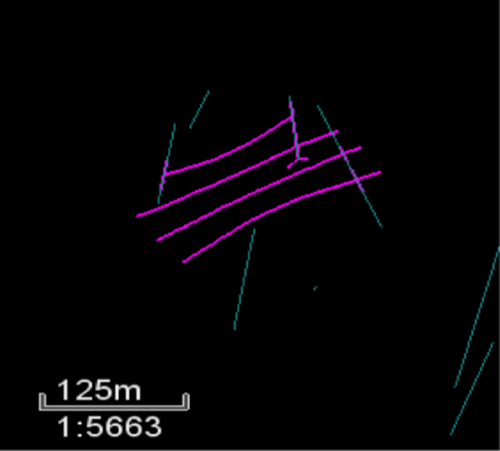
Simplistic fracture propagation.

**Figure 6:**
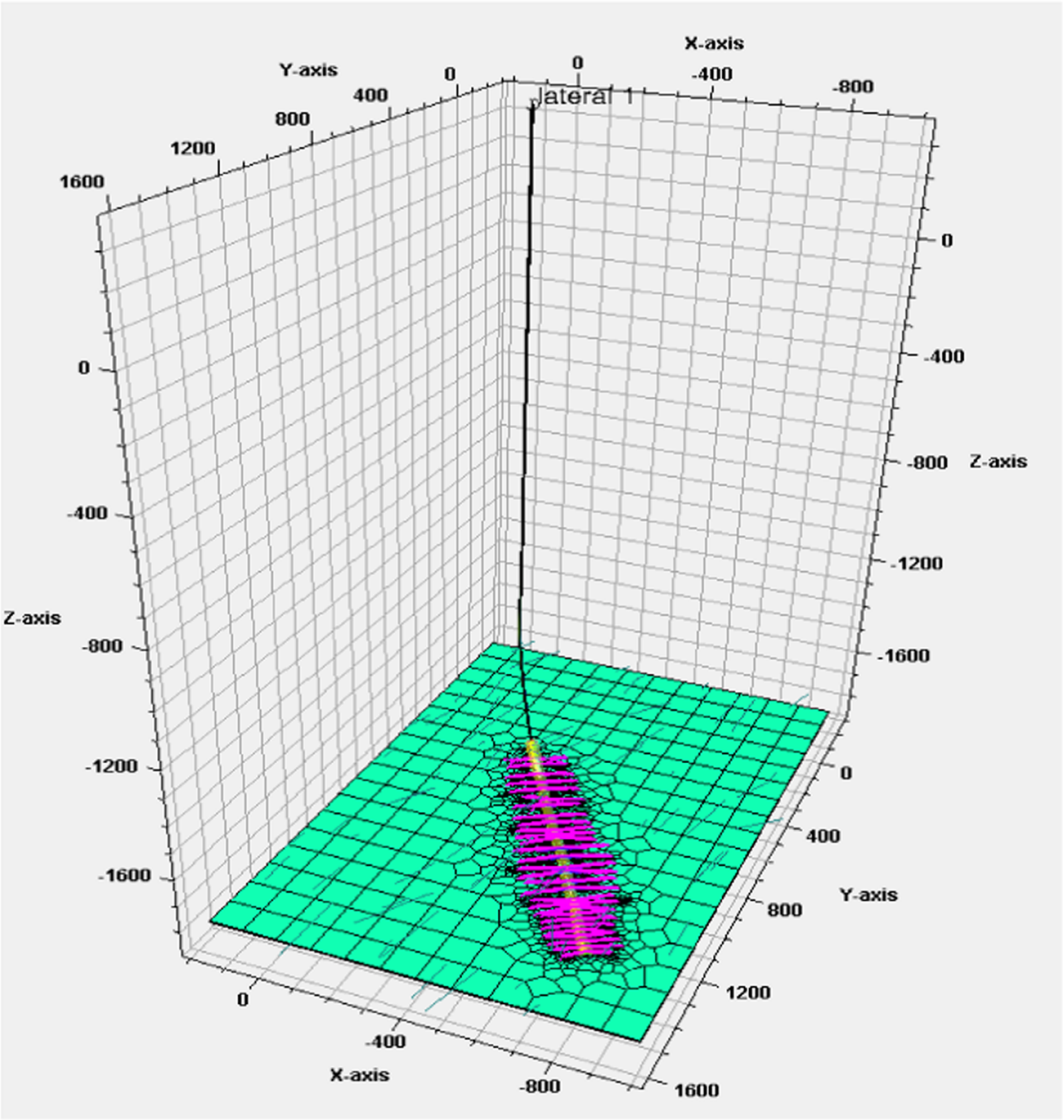
Fracture propagation response (zone indicated by green color).

**Figure 7:**
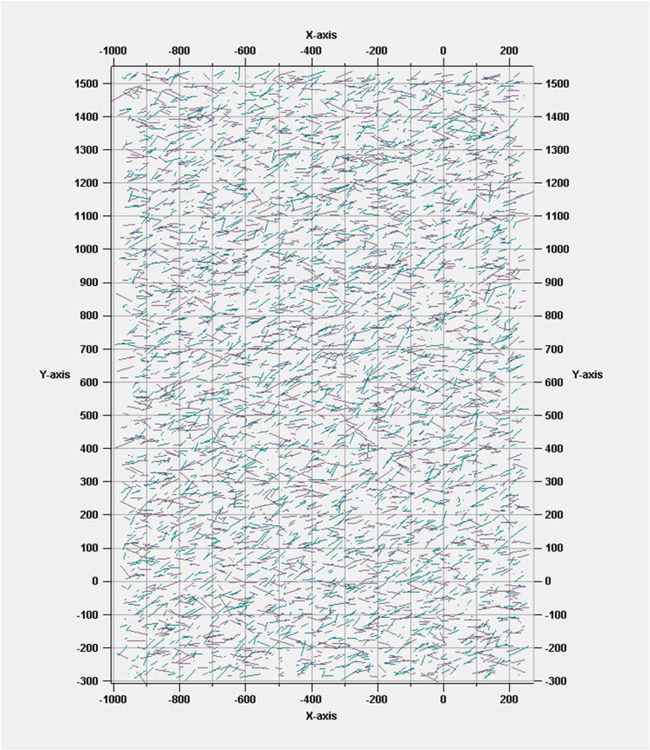
Discrete fracture network set (2D). Reprinted with permission from ref (9). Copyright 2020 Elsevier.

Figure 5 illustrates the fracture propagation behavior in the presence of a simple set of natural fractures. This was the starting point for this investigation to understand the hydraulic fracture propagation response in bounded scenarios. In this sample case, the different colors indicate the different types of proppants placed within the system and how the presence of a natural fracture may lead to improper placement, resulting in poor production. The cases were further extended by analyzing the fracture propagation response in a zone as shown in Figure 6, which served as the basis to screen compatible proppants and reservoir response. The effects of stress-shadowing and cross-fracturing were also considered, as illustrated in the figure. Additionally, multiple natural fracture sets were constructed to investigate the hydraulic fracture propagation response. Figure 7 shows a representative two-dimensional (2D) DFN set, which was incorporated into the simulation and validated successfully with the field data.

This examination is unique in numerous aspects. In addition to being one of the first simulation models with Middle Eastern field data, it also expands on the main findings presented by Suboyin et al., particularly with respect to water usage analysis.^9,27^ Over 346 simulation cases have been conducted for this investigation, and following are some key highlights.

(1) Construct and advance an adaptable simulator model to examine, identify, and quantitatively characterize the dominant fracture design parameters for the given reservoir conditions.
(2) Examine and analyze hydraulic fracture propagation for a candidate Middle Eastern tight gas reservoir in the presence of complex natural fractures.
(3) Analyze and quantitatively characterize the fracture propagation behavior to suggest an operational workflow tailored to the reservoir.
(4) Conduct a sensitivity analysis to identify key parameters influencing fracture geometry along with identifying potential design considerations and improving efficiency with respect to resource management.
(5) Investigation highlights the vital contribution of parameters such as fracturing fluid viscosity, proppant selection, and fracture aperture in regions with limited resources.
(6) Propose a unique operational and sustainable workflow to highlight the governing parameters for efficient water-management strategies for water-scarce and arid regions such as the Middle East.

**Figure 8:**
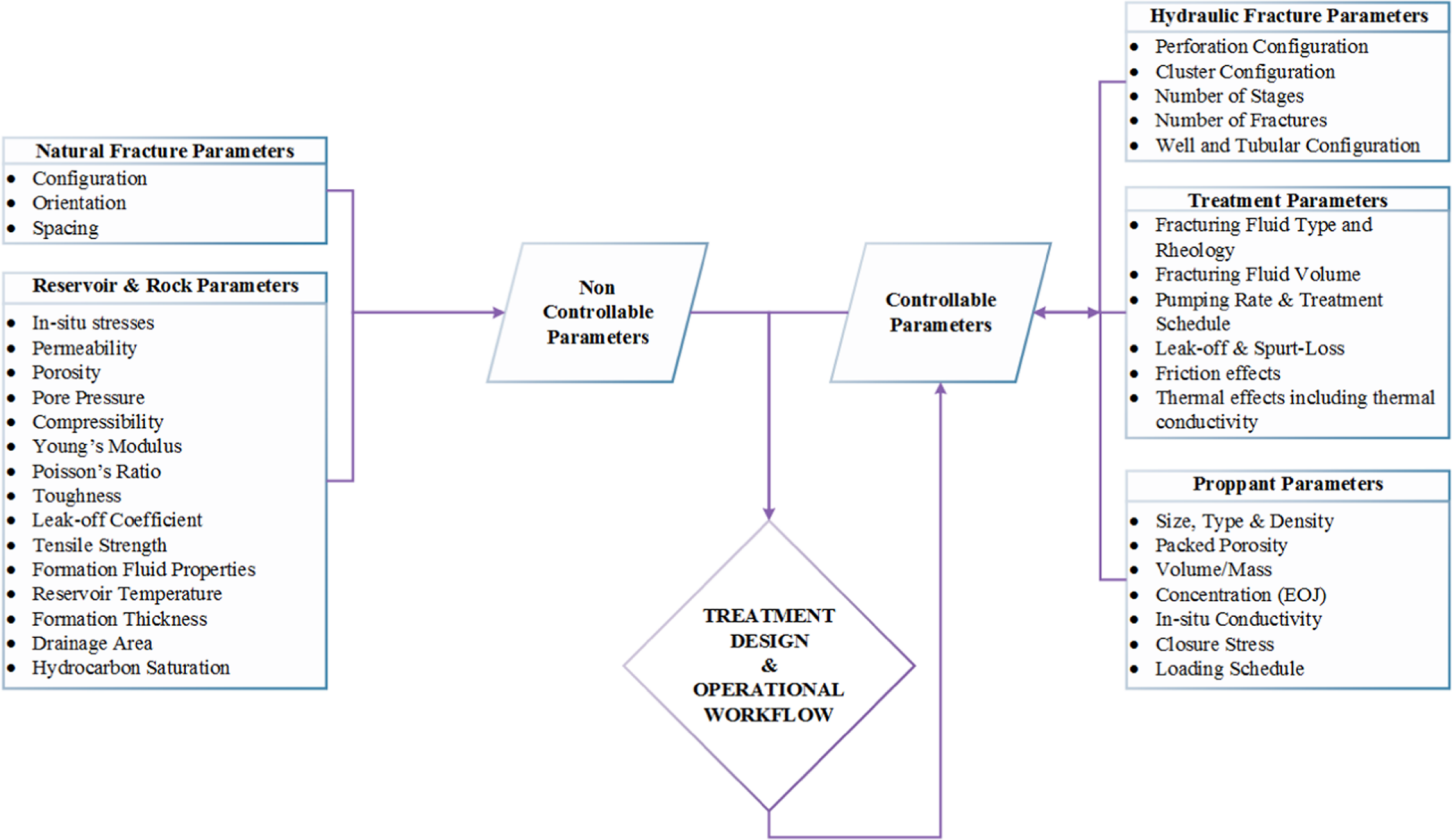
Parameters incorporated for the simulation and workflow.

Table 1 presents the input data for this investigation. Table 1 illustrates the fundamental input data incorporated for the constructed simulation model. The cumulative set of all parameters incorporated is depicted in Figure 8.

**Table 1:** Model Input Data: Summary

property	ranges	property	ranges
Young’s modulus (psi)	1 450 377–11 603 019	σ*V* (psi)	9282–9572
Poisson’s ratio	0.1–0.3	σ*h* (psi)	4206–6092
permeability (mD)	0.0001–1	σ*H* (psi)	4206–9572
porosity (%)	0–10	σ*H* – σ*h* (psi)	0–4351
fracture toughness (psi in.^1/2^)	910–1820	natural fracture length (ft)	50–200
tensile strength (psi)	290–870	natural fracture spacing (ft)	50–200
compressibility (1/psi)	2.07 ×10^14^–2.48 × 10^14^	natural fracture orientation (deg)	0–180
reservoir fluid viscosity (cP)	0.02	reservoir drainage area (acres)	80–100
reservoir pressure (psi)	2832–2930	total pay zone height (ft)	150–175
fracture spacing (ft)	16–1000	gas specific gravity	0.58
fracture width (in.)	0.00003–0.01	reservoir temperature (°F)	175–200

The input data was successfully validated after integration into the mode. It was compared and verified with field response, and they were found to be under reasonable limits (∼3% error). The limitations for this study were with respect to data sourcing and transparency. This was primarily an in-house investigation through internal data and case studies from operators and service companies within the region. Furthermore, with respect to the simulation model, the following were the key underlying assumptions.

(1) Hydraulic fracture height constraint: The zones and subzones were modeled, defined, and restricted to contain fracture propagation to the targeted zone. Fracture height containment was critical to accurately evaluate parametric influence.
(2) Fracture network constraint: The simulator was limited to a two-dimensional natural fracture network. A three-dimensional fracture network is often more representative of field conditions, and simulators with such a capability should be considered in further studies.
(3) Temperature constraint: The simulator was limited to account for highly accurate predictions with respect to the influence of temperature. Some of the results may vary as compared to field behavior.

## 3 Results and Discussion

Based on the systematic methodology constructed and the successive application of the methodology to field cases, the summary of results and discussion is depicted as follows. Figures 9, 10, 11, 12, 13, 14, 15, 16, and 8–17 further support the observations.

**Figure 9:**
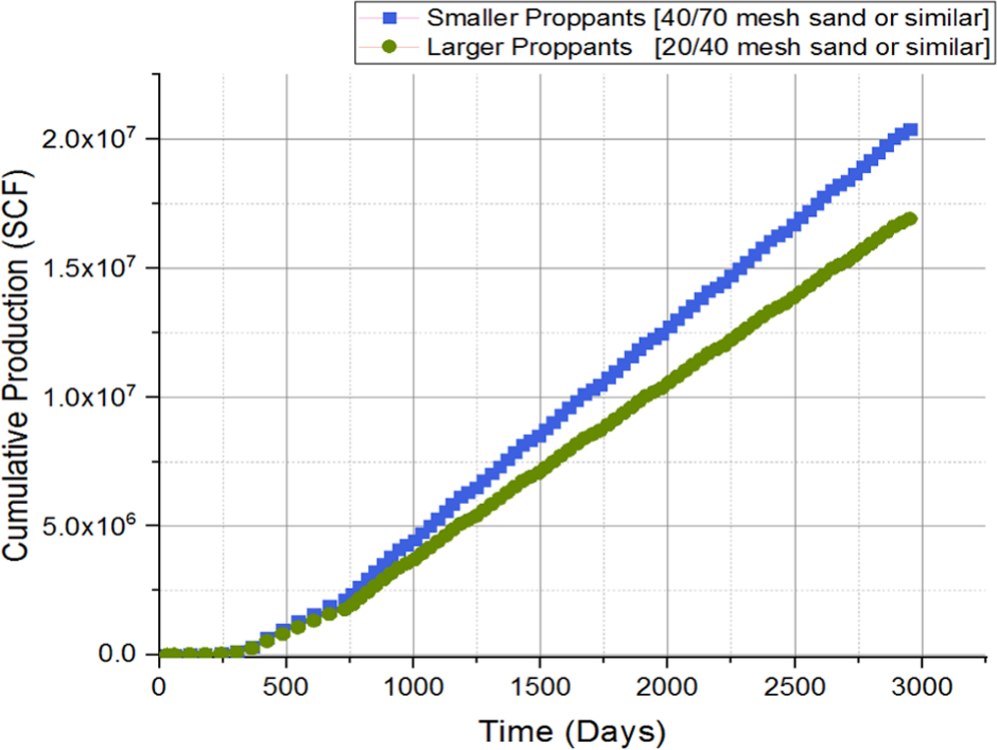
Cumulative production with respect to proppant type.

**Figure 10:**
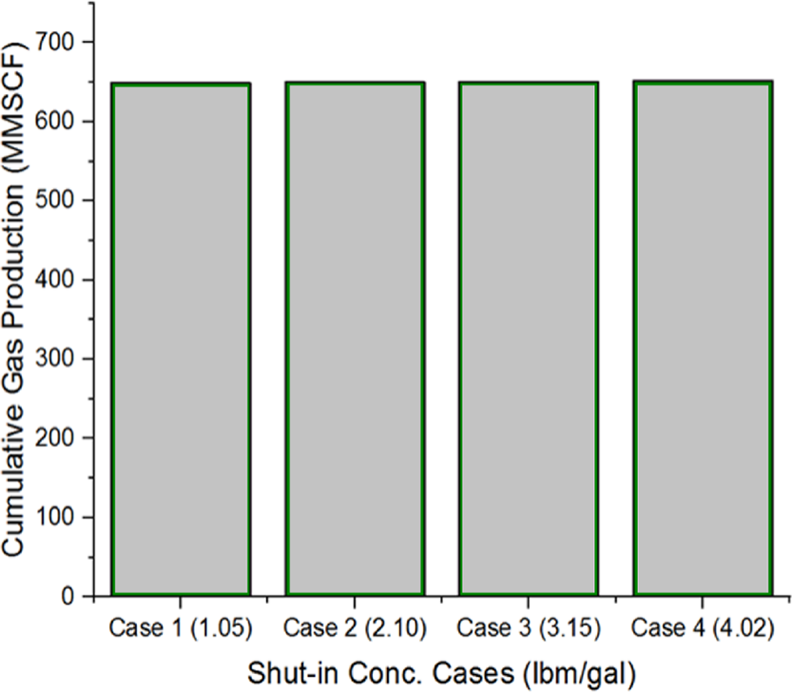
Change in cumulative gas production with respect to proppant concentration.

**Figure 11:**
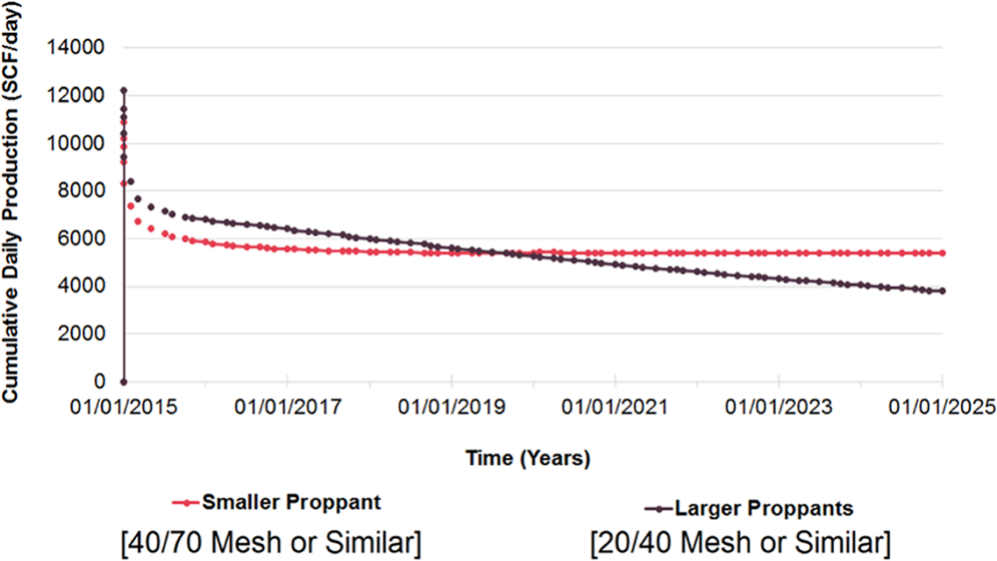
Change in daily gas production with respect to proppant sequence.

**Figure 12:**
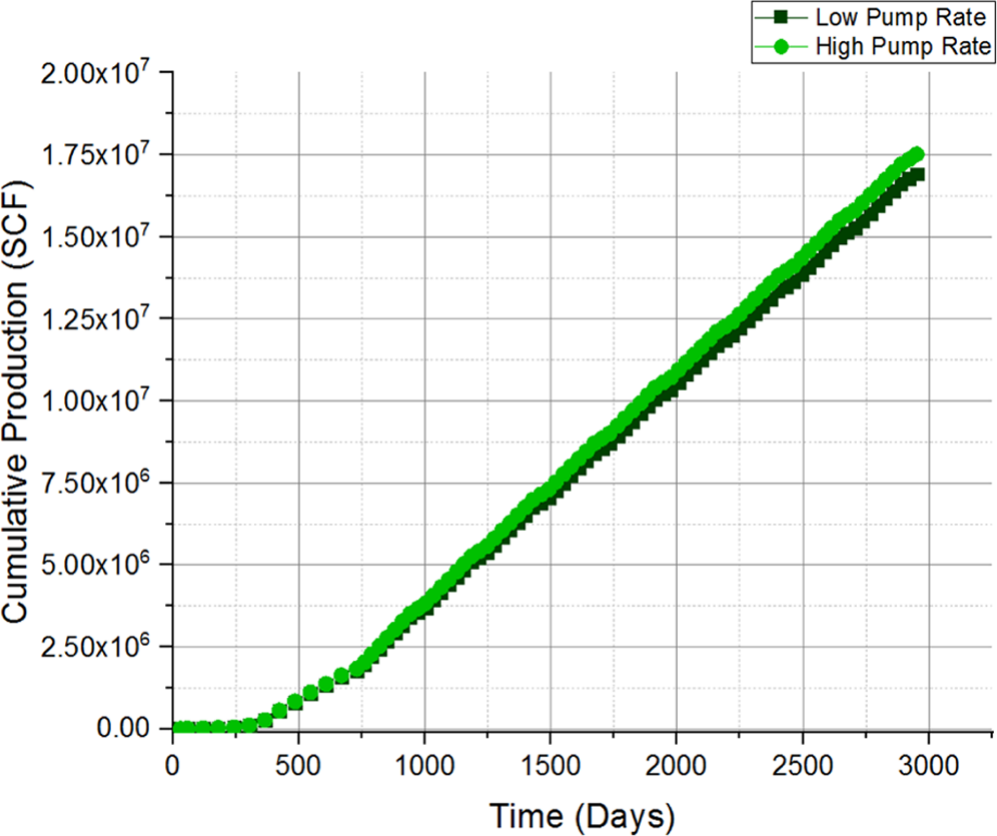
Change in cumulative gas production with respect to the pump rate.

**Figure 13:**
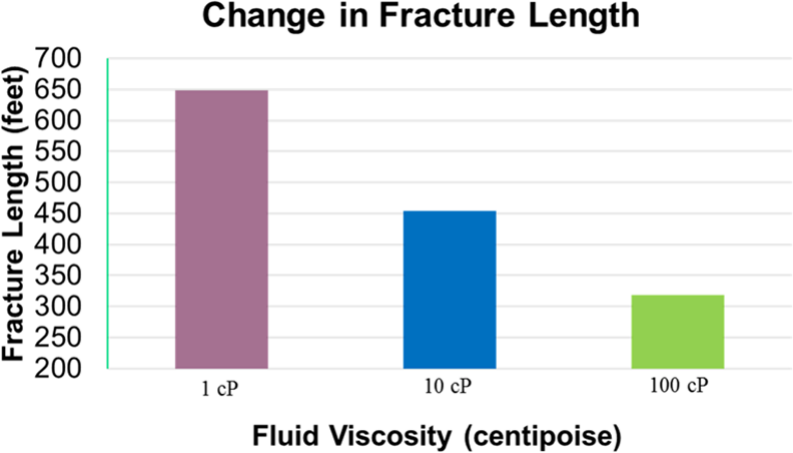
Change in fracture length with respect to fluid viscosity.

**Figure 14:**
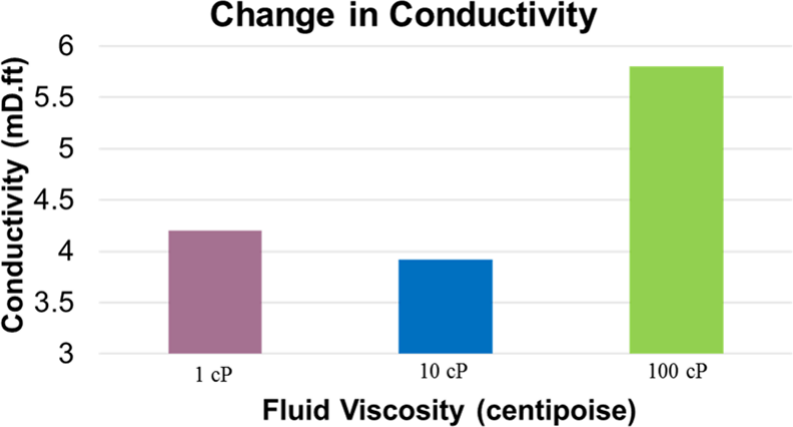
Change in fracture conductivity with respect to fluid viscosity.

**Figure 15:**
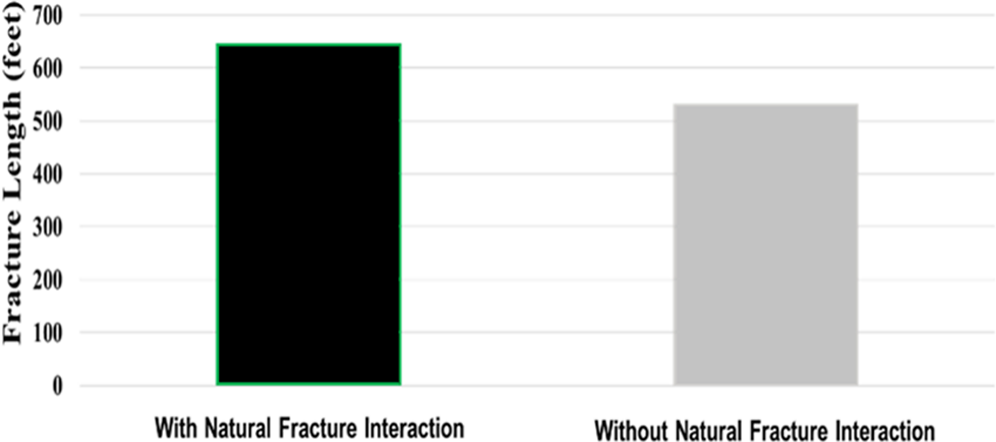
Change in fracture length with respect to natural fracture interaction.

**Figure 16:**
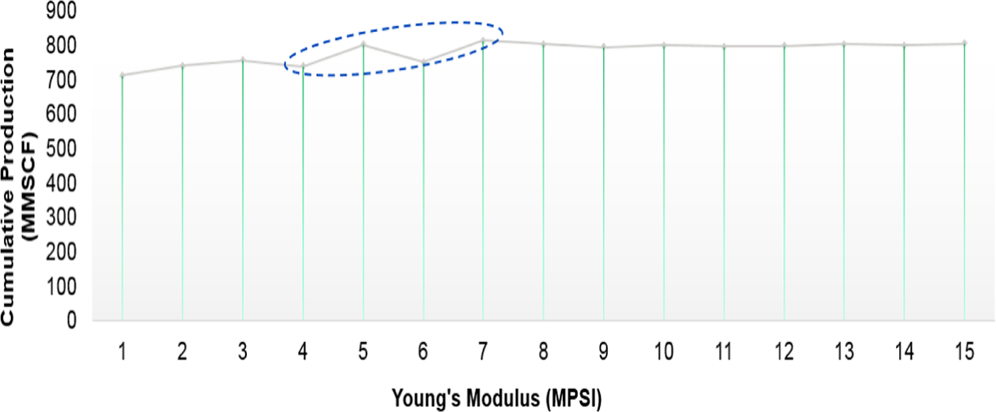
Change in cumulative production with respect to Young’s modulus.

**Figure 17:**
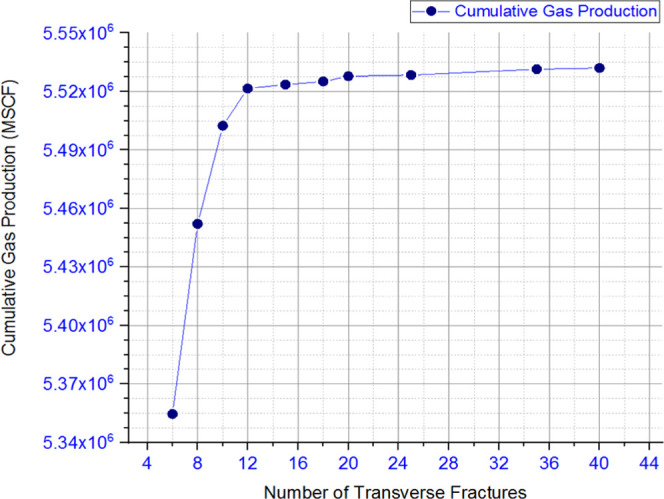
Change in cumulative gas production with respect to the number of transverse fractures (simplistic analysis).

The investigation conducted is segmented into two classifications.

(1) Controllable: Parameters that may be directly influenced, controlled, or directed such as hydraulic fracture design parameters, drilling activities, etc.
(2) Noncontrollable: Parameters that might not be directly influenced, controlled, or directed such as natural fracture distribution, etc.

**Table 2:** 

parameter	summary
proppant size	base case considered injection of one proppant being injected into a controlled operation; additional cases were constructed to examine the behavior with respect to different proppants
	it was observed that smaller proppants (40/70 mesh sand or similar) depicted a lower rate of production decline
	it was observed that larger proppants (20/40 mesh sand or similar) indicated a higher production, through the initial phase
	the total production was greater for larger proppants as depicted in Figure 9; related to proppant placement, settling, and flowback
	proppant can be tailored based on reservoir conditions for further optimization
proppant concentration	base case considered injection of one proppant with a predetermined fracturing fluid of suitable viscosity being injected into a controlled operation; additional cases were constructed to examine the behavior with respect to different proppant concentrations (1–4.02 ppg)
	proppant concentration as an individual parameter for a constrained base case did not contribute much to overall productivity as shown in Figure 10
	selection of fluid viscosity critical to proppant concentration, especially in zones where high concentration is required
	proppant concentration can be tailored based on reservoir conditions for further optimization
proppant sequence	base case considered injection of various proppants with a preset fracturing fluid of suitable viscosity being injected into a controlled operation; additional cases were constructed to examine the behavior with respect to different proppant sizes and sequences
	injection of smaller proppants (40/70 mesh or/and smaller) initially leads to an improvement in overall productivity as shown in Figure 11
	validated by field practices across the globe
pumping rate	base case considered injection of one proppant with a predetermined fracturing fluid of suitable viscosity being injected into a controlled operation; the pumping rate was varied (30–180 barrels per minute) to examine the behavior with respect to different pumping rates
	a direct linear growth in production was observed with respect to an increase in the pumping rate (30–150 barrels per minute) as shown in Figure 12; this is due to the fact that the flow rate was the only variable in the given simulation
	varying the fluid viscosity along with the pumping rate indicates that there can be a preferred pumping rate for a given reservoir for a given viscosity
fracturing fluid viscosity	base case considered injection of multiple proppants (40/70, 20/40, etc.) with a preset fracturing fluid being injected into a controlled operation; additional cases were constructed to examine the behavior with respect to different fracturing fluid viscosities
	vital to overall productivity and success of the operation
	variation with respect to induced fracture length and conductivity as illustrated in Figures 13 and 14
	simulations indicated that for the given constrained base case, using less viscous fluids induced a larger fracture length; with an increase in viscosity, there is an increase in fracture aperture coupled with a decrease in fracture propagation length, which leads to higher wellbore conductivity as depicted
	this is vital for regions that use slick water (low viscosity) as compared to engineered viscous fluids (high viscosity) for a particular formation
	fluid viscosity can be tailored based on reservoir conditions for targeting higher productivity

**Table 3:** 

parameter	summary
permeability	base case considered injection of one proppant into a controlled operation; additional cases were constructed to examine the behavior with respect to varying permeabilities
	for a controlled idealistic case, there is a linear increase in cumulate production with respect to an increase in permeability
natural fracture distribution	base case considered multiple proppants being injected into a controlled operation; additional cases were constructed to examine the behavior with respect to multiple sets of natural fractures as given in Table 1
	fracture propagation and response are reliant on natural fracture network distribution; the effect of fracture length is shown in Figure 15
	fracture length, fracture spacing, and natural fracture orientation alter the fracture propagation behavior and in turn affect the overall productivity
	can be detrimental to operations if proper consideration is not given
natural fracture density	base case considered multiple proppants being injected into a controlled operation; additional cases were constructed to examine the behavior with respect to multiple sets of natural fractures as given in Table 1
	natural fracture density significantly influences the fracture propagation behavior; simulations showed that an increase in fracture density resulted in an improvement in cumulative production
Poisson’s ratio	base case considered injection of multiple proppants into a controlled operation; additional cases were constructed to examine the behavior with respect to Poisson’s ratio ranges as given in Table 1
	within the ranges specified within the simulation, effect of Poisson’s ratio was limited under the same zone
	hence there is minimal influence with respect to cumulative production
Young’s modulus	base case considered injection of multiple proppants into a controlled operation; additional cases were constructed to examine the behavior with respect to Young’s modulus ranges as given in Table 1
	can be considered as a critical parameter and reference for fracture treatment design
	additional simulations with varying fluid properties and compatibility demonstrated productivity enhancements
	for the given set of data, there can be a region with a suitable value for Young’s modulus that may lead to higher productivity as shown in Figure 16; the importance and further behavior have been further explained in detail by earlier studies conducted by Suboyin et al.^27^
	coupling water-management parameters to optimize water usage further verifies the prominence of coupling Young’s modulus and identifies optimum ranges of fracturing fluid properties for a given reservoir

**Table 4:** 

parameter	key takeaway
hydraulic fracture aperture/width	hydraulic fracture width has a significant impact on overall productivity; in addition, this impact is greater than the impact of fracture length
hydraulic fracture length	even though there is an accompanying increase in cumulative production, the impact is less governing than an accompanying growth in fracture width
number of fracturing stages	the overall impact of number of stages was clearly indicated over the course of the 346 simulations conducted
	for a given reservoir and set of conditions, there is an optimum number of stages, beyond which the overall production does not increase substantially as shown in Figure 17
well placement	with the apparent effect and influence of the natural fracture network, well placement with respect to the network can significantly affect the overall production

**Figure 18:**
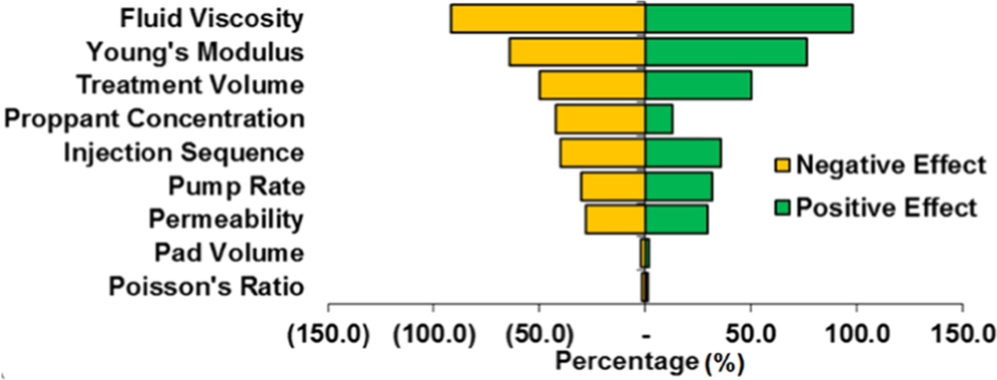
Sensitivity analysis.

To further analyze the significance of the parameters within the workflow, a sensitivity analysis as shown in Figure 18 along with a qualitative table (Table 5) was also constructed. This was achieved by evaluating the 346 simulations conducted in this study and relating each parameter influence and varying them in the ranges of (−50, −25, +25, and +50%) with respect to the cumulative production of the base case. Simulations demonstrate that fluid viscosity, treatment volume, proppant properties, and Young’s modulus are the most sensitive variables crucial to the overall productivity and water requirements for an operation. An in-depth analysis reveals that parameters such as proppant concentration can have a considerable negative effect for higher concentrations due to improper placement, proppant bridging, etc.

**Table 5:** Sensitivity Analysis (Qualitative)

rank	parameters	abs change (Δ%)
1	fluid viscosity	91
2	Young’s modulus	63
3	treatment volume	51
4	proppant size	47
5	proppant concentration	45
6	injection sequence	42
7	pumping rate	38
8	permeability	34
9	pad volume	3
10	Poisson’s ratio	1

**Figure 19:**
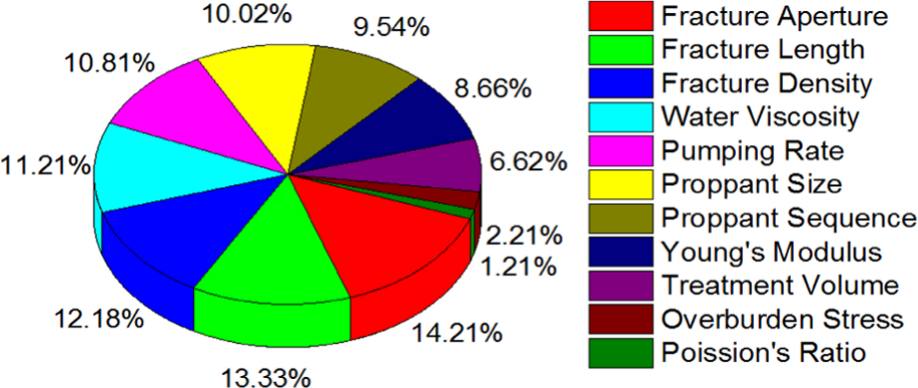
Parameter significance to overall productivity for the given reservoir.

Figure 19 further depicts a pie chart with the prominence of the key parameters. This aids in the identification of dominant parameters for an effective and efficient fracture design process for a given reservoir along with areas of potential concerns and complications. While analyzing these results, the interdependency of these parameters with other factors was apparent. This was also in line with the results from the simulations and internal case studies. This can greatly assist during the initial phase of the fracture treatment design process to identify the most suitable approach for a given set of data. It is imperative to highlight that the presented numbers do not depict a direct supremacy or priority over other listed parameters. This is related to the given set of data and may vary with cases.

The recent unconventional boom along with strict regulations also led to a paradigm shift in water-management strategies globally. For countries such as the United States, as there is no single water-management solution for a particular zone or play, there are significant variables and challenges for operators. Even today, there is a lack of holistic approach to assess the operational challenges in such regions. The lessons learned globally can greatly benefit future implementations where resources are constrained.

**Figure 20:**
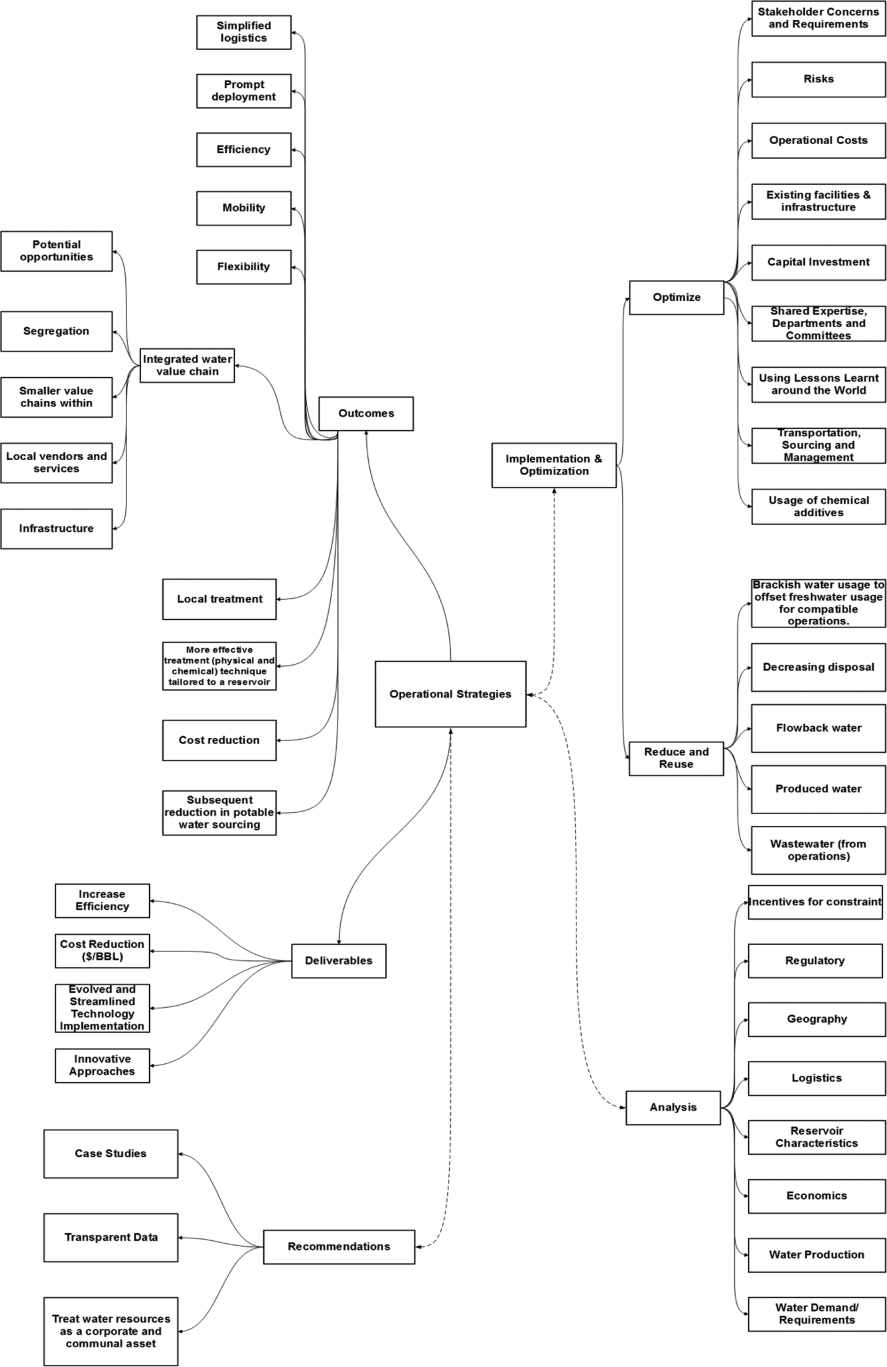
Proposed framework components.

Figure 20 depicts a flowchart generated based on a comprehensive review of internal field data and strategies implemented within the candidate field based on consultation with the operators in arid regions. This allowed us to identify areas of concerns, practical limitations, and potential opportunities to further streamline operations within the region. The investigations conducted and internal data emphasize the need to enhance implementation and optimization strategies within the region based on a comprehensive analysis of the given field conditions.

For instance, the constraints with respect to geography, reservoir, logistics, regulations, incentives, and economics play a critical role in overall analysis and the subsequent proposal of a tailored workflow based on the target objectives. Furthermore, as the workflow is predominantly based on water management, water requirements (including production, demand, and disposal) greatly influence the preliminary analysis. Furthermore, coupling the implementation and optimization techniques is key to the overall success of the workflow. This includes identification and successful execution of stakeholder requirements, regional risks, operational and capital expenditures, logistical constraints along with isolating silos within the organization for easier support, and shared expertise to address challenges. This leads to the critical part of the workflow, which defines the water-management strategies based on the target objective. For instance, for the given study, it was identified that based on the given conditions/constraints, reducing and reusing the produced water seems to be the most effective approach and has the potential to be expanded to other fields within the region. This includes increased usage of brackish water to offset freshwater requirements for compatible operations, treatment of produced water, flowback water, and wastewater along with minimal disposal.

One of the key outcomes for this study was identifying the potential for an integrated water value chain for the region. A disconnect observed within the domains, through internal data analysis, indicates potential opportunities for segregation within major operators and creation of smaller value chains through local vendors and service with the current infrastructure.

Additionally, if simplified logistics can be achieved with prompt deployment of the suggested techniques and technologies, this may result in an overall system that is more efficient and flexible. This would greatly assist in optimizing local treatment strategies, resulting in overall reduction in costs and potable water sourcing for local operations. This further led to identifying the key deliverables based on regional constraints. This includes enhanced and streamlined technology implementation of current treatment strategies for hydrocarbon operations and innovative approaches increasing the efficiency, resulting in reduced overall cost per barrel. This is also due to the fact that water used within current operations greatly adds to the overall cost per barrel for field operations in arid regions.

Selection of optimal strategies and innovative and tailored technologies can contribute significantly to the current market conditions, especially for arid regions such as the Middle East with their unique set of challenges and complexities. Previous investigations have already explored this earlier along with potential opportunities for the region.^9,27,28^ Further simulations on the fracturing model show how incorporating such a workflow along with the suggested considerations may aid in identifying the optimal number of transverse fractures for a given field with respect to resource management, as shown in Figure 17. As a result, this would assist in further reducing the cost and resources. Additional investigation on internal case studies revealed that accompanying variables such as stakeholder concerns and requirements, risks, operational costs, existing facilities and infrastructure, capital investment, shared expertise, departments and committees, case studies and lessons learnt around the world, transportation, and sourcing and management play a major role while defining strategies for a given region. Even though this may significantly add to the complexity, discretization of the existing methodologies along with integration of proven technologies by the multinational corporations (MNCs) into local operations can greatly assist the end-to-end components of a process. The dominant areas of such a chain would include water sourcing, treatment, reuse, transportation, storage, and disposal.

One of the recent evaluations conducted by IHS Markit presented a water-management cost model in the United States.^29^ This model analyzed costs for a hypothetical well in various scenarios mainly defined by the availability of freshwater and disposal wells. It was reported that they had a significant impact on well economics and water-management strategies for an operator. For arid and water-scarce regions, where water sourcing and disposal opportunities are constrained, strategic planning, conception, and implementation of a tailored water-management value chain can significantly contribute to the operating costs. It was suggested that recycling and reusing wastewater resources could still lead to considerable savings in capital expenditures (CAPEX) and operating expenses (OPEX) over time, even in regions where disposal rates may be high. For instance, it is reported that a representative Eagle Ford formation may need an average of 5 500 000 gallons of water per well.^6^ A rough internal analysis conducted on a candidate field in the Middle East, incorporating the proposed approach, shows the potential to reduce the required water for an operation by nearly 23% or around 1.3 million gallons. This was achieved through identifying a favorable viscosity for the fracturing fluid, selection of a compatible proppant, and optimizing water management for the given reservoir. In addition, further studies show there is potential to integrate management strategies along with the commercially viable and proven treatment methods that may be regionally specific. For example, to obtain the quality and quantity of water desired based on the water source (seawater, freshwater, produced water, etc.), desalination or reusing water sources may be an option. Furthermore, some regions also report that further transparency in data along with treating water resources as a corporate and communal asset can play a major role in future strategies in such regions.

## 4 Summary and Conclusions

In this research, a comprehensive investigation was conducted with respect to hydraulic fracture treatment design and fracture propagation in the presence of natural fractures. This allowed determining the distinct contribution and dominance of key parameters related to it. This can greatly assist in arid and water-scarce regions such as the Middle East where resources such as water and proppants are limited. In addition, the investigation indicates that there is strong potential for the petroleum industry to leverage its technology for an efficient water-management value chain for such regions. It is also to be highlighted that there is no bespoke solution to the best approach in such regions. However, a workflow tailor-made to the regional constraints may lead to the definition of more accurate, effectual, and practical strategies. This can also assist in enhancing existing methodologies and contributing to the overall process chain.

The key conclusions are as follows.

(1) An adaptable simulation model was constructed and advanced to examine, identify, and quantitatively characterize the dominant fracture design parameters for the given reservoir conditions along with water-management strategies.
(2) Hydraulic fracture propagation for a candidate Middle Eastern tight gas was examined in the presence of complex natural fractures.
(3) Quantitative characterization and design considerations presented can assist to create an operational workflow for sustainable resource management tailored to the Middle Eastern tight gas reservoirs.
(4) Relative significance along with a sensitivity analysis further highlights the relevance of the dominating parameters to fracture propagation and geometry. This can contribute to improving current methodologies while improving efficiency with respect to resource management.
(5) Fracturing fluid viscosity, proppant selection, and fracture aperture play a major role in regions with limited resources. An in-depth analysis of these parameters with respect to a reservoir can provide a better insight into the predicted response and potential to enhance fracturing operations for arid regions. This is crucial in the Middle East, renowned for its highly heterogeneous reservoirs.

## Appendix

As per the literature,^23−25^ the equation for mass conservation for an incompressible slurry that is to be pumped into the fracture was represented as eq 1. Under eq 1, we have

5

6

7

wherein *t* is the time required for fracture leak-off area creation, *V*_f_ is the volume of fracture, *V*_l_ is the fluid loss, *V*_sp_ is the spurt loss, *t*_p_ is the pumping time, *C* is the total leak-off coefficient, *A* is the leak-off area, *S*_p_ is the spurt-loss coefficient, and α_a_ is the leak-off parameter.

For any hydraulic fracturing treatment, it is critical to consider leak-off fluid loss, pre and post pumping. This was expressed as eqs 2 and 3.^25^ Under eq 3, θ is dimensionless time and is represented as

8

wherein *t* is the time required for fracture leak-off area creation and *t*_p_ is the pumping time.

With regard to the mass continuity equation with respect to flow rate per unit length (*q* = *vW*), we have eqs 9 and 10(25)

9

where

10

wherein *v* denotes Poisson’s ratio, *q* is the change in flow rate, *q*_L_ is the injection flow rate, *W* is the fracture width, *x* is the coordinate along the length of the fracture, *y* is the coordinate perpendicular direction of fracture, and *z* is the coordinate along the vertical.

The equation of motion or the widely known equation for momentum with respect to steady-state flow is expressed as eq 11(25)

11

where

for laminar flow and

*f* = *f*(*Re*, ε) for turbulent flow, wherein *f* is the Darcy friction factor, *Re* is the Reynolds number, and ε is the relative wall toughness.

## Nomenclature

*A*: area (leak-off)
*P*: change in pressure
*z*: coordinate (vertical)
*x*: coordinates (along the length of fracture)
*y*: coordinates (perpendicular direction of fracture)
*f*: Darcy friction factor
ρ: density
θ: dimensionless time
*V*_*l*_: fluid loss
*G*: generalized function (fluid loss)
Γ: generalized function (influence)
*H*_ξ_: half-height (characteristic)
*q*: injection flow rate (general)
*q*_*L*_: injection flow rate (liquid)
C: leak-off coefficient (total)
α_*a*_: leak-off parameter
α_*τ*_: leak-off parameter (at fracture)
α_*c*_: leak-off parameter (at pumping)
π: pi
v: Poisson’s ratio
Φ: porosity
ε: relative wall toughness
α_c_2__: reservoir compressibility and viscosity
*Re*: Reynolds number
*V*_sp_: spurt fluid loss
*S*_p_: spurt loss coefficient
*t*: time
τ: time (fracture leak-off area creation)
*t*_*P*_: time (pumping)
*V*_*f*_: volume (fracture)
*W*: width of fracture
